# Novel approach to visualize the inter-dependencies between maternal sensitization, breast milk immune components and human milk oligosaccharides in the LIFE Child cohort

**DOI:** 10.1371/journal.pone.0230472

**Published:** 2020-04-21

**Authors:** Loris Michel, Maya Shevlyakova, Ellen Ní Cléirigh, Erik Eckhardt, Sebastien Holvoet, Sophie Nutten, Norbert Sprenger, Antje Körner, Mandy Vogel, Chiara Nembrini, Wieland Kiess, Carine Blanchard

**Affiliations:** 1 Nestlé Research, Vers-chez-les-Blanc, Lausanne, Switzerland; 2 Nestlé Development Centre Nutrition, Askeaton, Ireland; 3 Faculty of Medicine, LIFE Leipzig Research Center for Civilization Diseases, University of Leipzig, Leipzig, Germany; UAMS/ACHRI/ACNC, UNITED STATES

## Abstract

**Background:**

Numerous studies have shown that specific components of breast milk, considered separately, are associated with disease status in the mother or the child using univariate analyses. However, very few studies have considered multivariate analysis approaches to evaluate the relationship between multiple breast milk components simultaneously.

**Aim:**

Here we aimed at visualizing breast milk component complex interactions in the context of the allergy status of the mother or the child.

**Methods:**

Milk samples were collected from lactating mothers participating in the Leipziger Forschungszentrum für Zivilisationskrankheiten (LIFE) Child cohort in Leipzig, Germany. A total of 156 breast milk samples, collected at 3 months after birth from mother/infant pairs, were analyzed for 51 breast milk components. Correlation, principal component analysis (PCA) and graphical discovery analysis were used.

**Result:**

Correlations ranging from 0.40 to 0.96 were observed between breast milk fatty acid and breast milk phospholipids levels and correlations ranging from 0 to 0.76 between specific human milk oligosaccharides (HMO) were observed. No separation of the data based on the risk of allergy in the infants was identified using PCA. When graphical discovery analysis was used, dependencies between maternal plasma immunoglobulin E (IgE) level and the breast milk immune marker transforming growth factor-beta 2 (TGF-ß2), between TGF-ß2, breast milk immunoglobulin A (IgA) and TGF-ß1 as well as between breast milk total protein and birth weight were observed. Graphical discovery analysis also exemplifies a possible competition for the fucosyl group between 2’FL, LNFP-I and 3’FL in the HMO group. Additionally, dependencies between immune component IgA and specific HMO (6’SL and blood group A antigen tetraose type 5 or PI-HMO) were identified.

**Conclusion:**

Graphical discovery analysis applied to complex matrices such as breast milk composition can aid in understanding the complexity of interactions between breast milk components and possible relations to health parameters in the mother or the infant. This approach can lead to novel discoveries in the context of health and diseases such as allergy. Our study thus represents the first attempt to visualize the complexity and the inter-dependency of breast milk components.

## Introduction

Human breast milk is the gold standard for infant nutrition, providing optimum nutrition and immune protection for healthy growth and development [1]. As early as 1898, the British Medical Journal published a study associating breast milk components with childhood morbidity and mortality [2]. In 1900, the complexity of breast milk composition and the changes in composition with the mother's health status were discussed [3]. Since then, numerous reports have described the effect of breast milk composition on atopic or allergic diseases in infants, as well as the effect of environmental factors such as maternal diet or lifestyle behaviours on breast milk composition [4–6].

Currently, the literature has yet to reach a consensus on whether any breast milk component or a specific profile for breast milk composition is associated with allergy risk, allergic sensitization, or allergic disease in the infant [7, 8]. Numerous studies have associated breast milk levels of transforming growth factors (TGF) such as TGF-ß1, TGF-ß2 and/or levels of immunoglobulin (Ig) A with allergy in infants or mothers [9–11], yet a recent review suggests that the data were not robust enough to reach a conclusion on TGF-ß1 and 2 [12]. Similarly, breast milk fatty acids such as polyunsaturated fatty acid (PUFA) and polyunsaturated fatty acid (MUFA) levels or ratio have been found inversely associated with allergy risk in the infants while others did not found any association [13]. Finally, 3 recent studies have suggested a possible association between human milk oligosaccharides (HMO) and atopic dermatitis [14, 15], milk allergy [14] or allergic sensitization [16] in the infants. Interestingly, the latter study did not found direct positive or negative associations between HMO levels and allergic sensitization using univariate analysis but rather identified that overall profiles should be considered when examining the health effects of HMO and the risk of sensitization in infants [16]. Besides the possible confounding environmental factors, the study design, methodology, and sample size, these conflicting results in the literature also emphasized the complexity of the association between breast milk composition and allergy, and suggest that univariate analysis in complex data set [17] or in a dynamic fluid, like breast milk, may be limited due to underlying interactions amongst components.

To date, very few studies have considered multivariate analysis approach to evaluate the relationship between multiple breast milk components simultaneously and with disease risk, and even fewer in the context of allergy [13, 16]. Visualization of complex interactions is thus an interesting method to generate new hypothesis and discoveries in a complex matrix like breast milk. To our knowledge, none used graphical causal discovery algorithms to visualize the possible direct and indirect interaction between breast milk components.

In this short communication, we illustrate hidden breast milk component interactions that might affect the association with clinical parameters like allergic sensitization in the mother. Multivariate analysis approach was used to assess the interactions among 51 different breast milk components, along with some of the main confounders which influence breast milk composition and allergic sensitization in the mother and the infant or allergic outcomes in the infant. Breast milk samples used in the analysis were collected from a group of mothers enrolled in the Leipziger Forschungszentrum für Zivilisationskrankheiten (Leipzig Research Center for Civilization Diseases) (LIFE) Child study in Germany [18].

## Methods

### Breast milk cohort

This cohort of 156 mother/infant pairs were selected from the LIFE Child study (5). When the project started in March 2015, at that time, 333 mother-infant pairs were included in the LIFE Child cohort since 2011. Of these 333 pairs, 237 mothers (75%) provided breast milk samples at 3 months, 156 pairs were selected due to the completion of the allergy questionnaires in the mother and or the child. The number of subjects in the different groups is summarized in Table 1 and S1 Fig. While the sample size is small (n = 156) allergy prevalence was in expected ranges for the general European/German infant population. The selection of the samples was based on the availability of breast milk samples at three months of life along with the completion of the allergy questionnaires in mothers at any time during the study and/or infants in the first year of life of the infant. For the purpose of the study, maternal allergic sensitization was defined by the total IgE level in plasma greater than 127 KU/L [19] and allergy defined as self-reported asthma, rhinitis, atopic dermatitis eczema and, and / or allergic reactions to specific food (with vomit, nausea, diarrhea, exacerbation of eczema or asthma symptoms) in the mother. For the purpose of this study, infant allergic sensitization was defined as a total plasma IgE greater than 35 kU/L and 53 kU/L at 6 and 12 months respectively [19], and allergy risk as a positive answer to the questions “did a doctor ever diagnose food allergy in your child” “Did your child ever have eczema / atopic dermatitis” or “did your child suffer from recurrent rashes associated with pruritus during more than 15 days” at 3, 6 months or at one year. Mother and child total plasma IgE was quantified using ImmunoCAP© Phadia Laboratory Systems technology (Phadia AB, Uppsala, Sweden). In this cohort, allergy and confounders such as socio-economic status were obtained via various questionnaires. Confounders were identified based on available literature associating allergy and breast milk components and were chosen based on available data (delivery mode (delivery), child gender (gender), child weight at birth (weight), exclusively breastfeeding at 3 months (breastfeed), socioeconomic status (ecoStatus) and number of siblings (siblings). We used this cohort as an exploratory cohort. Breast milk was expressed, collected and stored at −80°C from lactating mothers the third month postpartum as previously described [20]. Only one mother/child pair was excluded due to missing values for the breast milk components levels (>50%). The study was designed in accordance with the Declaration of Helsinki and under the supervision of the Ethics Committee of the University of Leipzig (Reg. No. 264-10-19042010). All mothers included were informed and provided their written informed consent to participate. LIFE Child study is registered in ClinicalTrials.gov under the clinical trial number: NCT02550236. [18] The legal requirements and the given informed consent do not allow public sharing of the dataset. Interested researchers can contact LIFE Child (research@life-child.de) for further information on access to the dataset PV-0195.

**Table 1:** Baseline demographics.

Mothers	N	Mean	Median	SD	Min	Max
Age at birth (years)	156	30.57	30.61	4.36	22.60	42.12
IgE (36 weeks)	129	193.16	37.90	734.62	0.90	6026.00
**Allergic status out of 156 Mothers**	**Count**	**Percentage**				
Non-allergic	42	26.92				
Atopic dermatitis or sensitization (>127KU/L)	53	33.97				
Atopic dermatitis only	22	14.10				
Sensitization only (>127KU/L)	36	23.08				
Asthma	10	6.41				
Allergic rhinitis	26	16.67				
Food allergy	10	6.41				
Any of the above	60	38.46				
**Infants**	**N**	**Count**	**Percentage**			
Male	156	82	52.56			
Caesarian delivery	155	24	15.48			
Exclusive breastfeeding at 3 months	138	121	87.68			
Dog keeping	148	13	8.78			
Cat keeping	148	24	16.22			
Smoke exposure in house	126	10	7.94			
	**N**	**Mean**	**Median**	**SD**	**Min**	**Max**
Gestational age (weeks)	153	39.73	40.14	1.64	30.71	41.86
Birth weight (g)	156	3442.29	3435.00	514.52	2230.00	5035.00
Duration of exclusive breastfeeding (months)	150	4.52	5.00	1.92	0.00	13.00
Income (level 1 to 13)	148	8.49	9.00	2.60	3.00	13.00
IgE level at 6 months	57	20.79	2.00	106.52	<0.1	803.00
IgE level at 1 year	47	52.08	8.00	155.90	0.30	1002.00
**Children allergic status out of 156 included**	**Count**	**Percentage**				
Non-allergic	40	25.64				
Atopic dermatitis (Diagnosed or Incidence recurrent itchy rashes) or allergic sensitization (IgE>30 at 6 months n = 5 or >53KU/L at 1 year n = 8)	36	23.08				
Atopic dermatitis (Diagnosed)	20	12.82				
Atopic dermatitis (Incidence >15 days of recurrent itchy rashes)	18	11.54				
Rashes (diaper rashes excluded)	56	35.90				

### Breast milk component level assessment

In total, 51 breast milk components were measured using the following technics: enzyme-linked immunosorbent assay (ELISA) for breast milk TGF-β1, TGF-β2 using Quantikine ELISA Kit (R&D systems ® Inc., Bio-Techne AG, Zug, Switzerland), IgA (Bethyl Laboratories, Montgomery, Texas, USA), beta-lactoglobulin (BLG) RIDASCREEN® ELISA (R-Biopharm AG, Darmstadt, Germany) and by colorimetric assay for breast milk choline MAK056 (Sigma-Aldrich, Buchs SG, Switzerland). Breast milk folate was quantified using the Siemens (Siemens), human milk oligosaccharides (HMO) 2’ Fucosyllactose (2FL), 3’ Fucosyllactose (3FL), 3’ Sialyllactose (3SL), 6’ Sialyllactose (6SL), Lacto-N-tetraose (LNT), Lacto-N-neotetraose (LNnT), Lacto-N-fucopentaose V (LNFP-V), Lacto-N-neofucopentaose (LNnFP)) Blood group H antigen pentaose type 1 (LNFP-I), blood group A antigen tetraose type 5 (PI-HMO) were selected because there are the ten HMO that can be quantified with their own standards and were measured by UHPLC as previously described [21]. Breast milk fatty acids (6:0, 8:0, 10:0, 12:0, 14:0, 16:0, 16:1.n-7, 18:0, trans-18:1, 18:1.n-9, 18:1.n-7,18:2.n-6, 18.3.n-3, 18.3.n-6, 20:0, 20:1.n-9, 20:2.n-6, 20:3.n-6, 22:1.n-9, 20:4.n-6, 20:5.n-3,22:6.n-3, 24:0, 24:1.n-9) were measured using previously published methods [22, 23]. Total fatty acids (total Fat) levels were calculated as the sum of individual fatty acids. Breast milk MUFA, PUFA, omega-3 conversion rate(N3cr), omega-6 conversion rate (N6cr) were calculated measures based on fatty acids data. The N3cr rate was calculated by dividing the docosahexaenoic acid (DHA) by the alpha-linolenic acid (ALA) content (DHA:ALA). The N6cr ratio was calculated by dividing the arachidonic acid (ARA) by the linoleic acid (LA) content (ARA:LA) [24]. Sphingomyelin (SM), phosphatidylcholine (PC), phosphatidylethanolamine (PE), phosphatidylinositol (PI), phosphatidylserine (PS), were also measured by High-performance liquid chromatography (HPLC). Total phospholipids (TotalPhos) was calculated as the sum of phospholipids. Total protein (TotalProt) levels were assessed using Pierce BCA Protein Assay Kit (Thermo Scientific, Rockford, Illinoi, USA). Vitamin A was assessed using iCheck fluoro kit (Bioanalyt, Teltow, Germany). As such, up to 51 breast milk components and 2 lipid ratios were used for the analyses.

### Statistical analysis

Statistical analyses were performed with the statistical software R, version 3:3:1. In the exploratory analysis, Pearson and Spearman’s correlations were estimated and their pairwise significance evaluated. For the principal component (PCA) and correlation analyses all the loading extractions as well as the projection of variables and observations on the principal components were implemented with help of the R package prcomp [25]. A total of 3 sets of analyses were performed 1) Raw PCA: PCA analysis with raw data of breast milk components quantification and without some of the aggregated variables (introducing evident collinearity in the data). 2) Reduced PCA: PCA analysis with a reduced number of variables suggested by the results of the first PCA. Namely, all individual fatty acids and phospholipids were grouped in the new PCA by their respective total amounts. 3) PCA with mothers IgE values: PCA analysis as conduced in 2) with IgE values from mothers as added variable to see how mothers IgE correlate with the variables from the second PCA.

### Graphical causal discovery algorithm

We applied a causal discovery algorithm named pc (Peter-Clark) with the help of the R package pcalg [25] and based on causal and graphical inference. The algorithm looks iteratively for conditional independence relations (based on statistical tests) between variables in the dataset by increasing at each step the size of the conditioning set. Since we are facing both continuous and binary variables, we had to adapt our tests to the nature of the data. We tested statistical independence of two variables x and y (for example x = total fatty acids and y = weight) conditioned on a set of variables S (for example S = gender; TGF-β1) then we follow the branching: 1). If S is void (test of marginal independence): if x and y are both binary, we use Fisher-exact test (function fisher.test in R) to test the association between the variables; if one variable is binary and the other continuous, we test for a difference of mean of the continuous variable with respect to the two groups induced by the binary one using the t-test (function t.test in R); if both are continuous, we use the correlation test (function cor.test in R). 2) If S is not void (test of conditional independence): we fit either a linear regression model or a generalized binomial regression model (functions lm and glm.fit in R) to the variables x and y depending on their respective nature with covariates being interactions between variables from the set S. So that we adjust x and y for these predictors separately. We then extract the residuals (and generalized residuals for the binomial regression) from the respective models and use the correlation test on these residuals to assess conditional independence of x and y. Moreover, since our analysis was exploratory, we did not correct for multiplicity. Concerning missing data, out of the initial pool of 156 mother/child pairs, complete cases for the 51 milk components amount to 148 (this number reduces to 130 if we consider milk components and the 5 confounders used in the analysis). In average, out of the 56 variables (51 milk components and 5 confounders) 55 are measured per mother/child pair. One mother/child pair presenting 68% of missing values was removed from the analysis set (all other pairs have at most 2 missing values out of 56 variables). The remaining missing values were imputed by the observed median per variable resulting in a final analysis set of 155 mother/child pairs. As for the confounders concerned, we did not include the number of older siblings as a variable in the analysis, due to the too high number of missing values (more than 50% of the observations).

## Results

When the project started in March 2015, 333 mother-infant pairs were included in the LIFE Child cohort. Of these 333 pairs, 237 mothers provided breast milk samples at 3 months, 156 pairs were selected due to the completion of the allergy questionnaires in the mother and or the child (S1 Fig). The average mother age at birth was 30.57 years and the median total IgE level was 37.90KU/L. A total of 42 mothers reported no allergic disease, and a total of 60 mothers were reporting at least one type of allergic disease or had a total IgE level >127KU/L as seen in Table 1. A total of 52.56% of the infants were males, infants were born on average during the 40^th^ week of pregnancy. Total IgE level was quantified in n = 57 infants at 6 months and n = 47 infants at 1 year with median values of 2 and 8KU/L respectively. To determine the risk of allergy in the infants a combination of symptoms involving a possible skin impairment, or a high sensitization level based on total IgE were considered (S1 Fig). To be considered at risk of allergy, infants needed to have a diagnosed AD, an incidence of long episodes (>15 days) of recurrent itchy rash reported by the mother, or a total IgE level >to 30 KU/L at 6 months and > 53KU/L at 1 year. Five infants were above the total IgE cut off level at 6 months and n = 8 infants at 1 year. A total of 40 infants was classified as low risk of allergy. Some of the leading confounders including delivery mode, gender, birth weight, breastfeeding exclusivity and duration, economic status, and sibling number were recorded (Table 1).

### Identification of correlation between breast milk components

**Fig 1:**
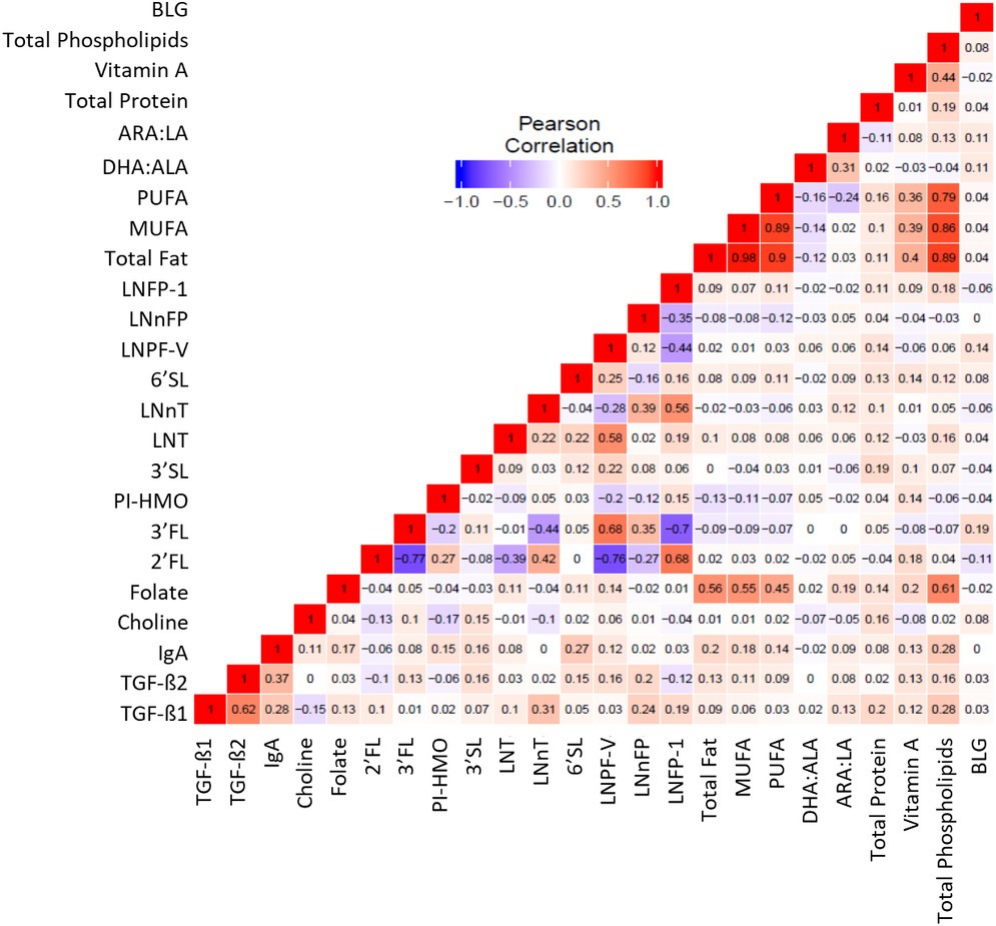
Correlation matrix of breast milk components. Pearson correlations represented by a color gradient based on the r coefficient are shown for the 24 selected breast milk components.

Pearson and Spearman’s correlations both showed positive correlations between breast milk fatty acids and phospholipids, as well as positive and negative correlations between specific HMO (S2 Fig). The correlation coefficient within the fatty acids and phospholipids ranged from 0.40 to 0.96. Recent findings suggest that breast milk fatty acid levels are not associated with allergic disease risk. [13, 26] Fatty acids thus were grouped to better identify other weaker correlations, and total fatty acids, polyunsaturated fatty acid (PUFA), monounsaturated fatty acid (MUFA) and MUFA and PUFA conversion ratios (DHA:ALA and ARA:LA) were then conserved (Fig 1). As previously shown, we confirmed the correlation between the immune factors TGF-β1 and TGF-β2 with an r = 0.62, (Fig 1). Correlations between IgA and TGF-β1 or IgA and TGF-β2 were r<0.40. Some correlations between HMO were also found as significant (p<0.05) (S2 Fig), and these included the following: 2’-fucosyllactose (2FL) was inversely correlated with 3-fucosyllactose (3-FL) and lacto-N-fructopentaose V (LNFP-V) (r = -0.77 and -0.76, respectively) and positively correlated with lacto-N-fuctopentaose I (LNFP-I) (r = 0.68); 3-FL was positively correlated with LNFP-V and inversely with LNFP-I (r = 0.68 and -0.7, respectively). Other low and moderate correlations were also seen between other components as shown in Fig 1A.

### Principal component analysis in allergic and non-allergic individuals

**Fig 2:**
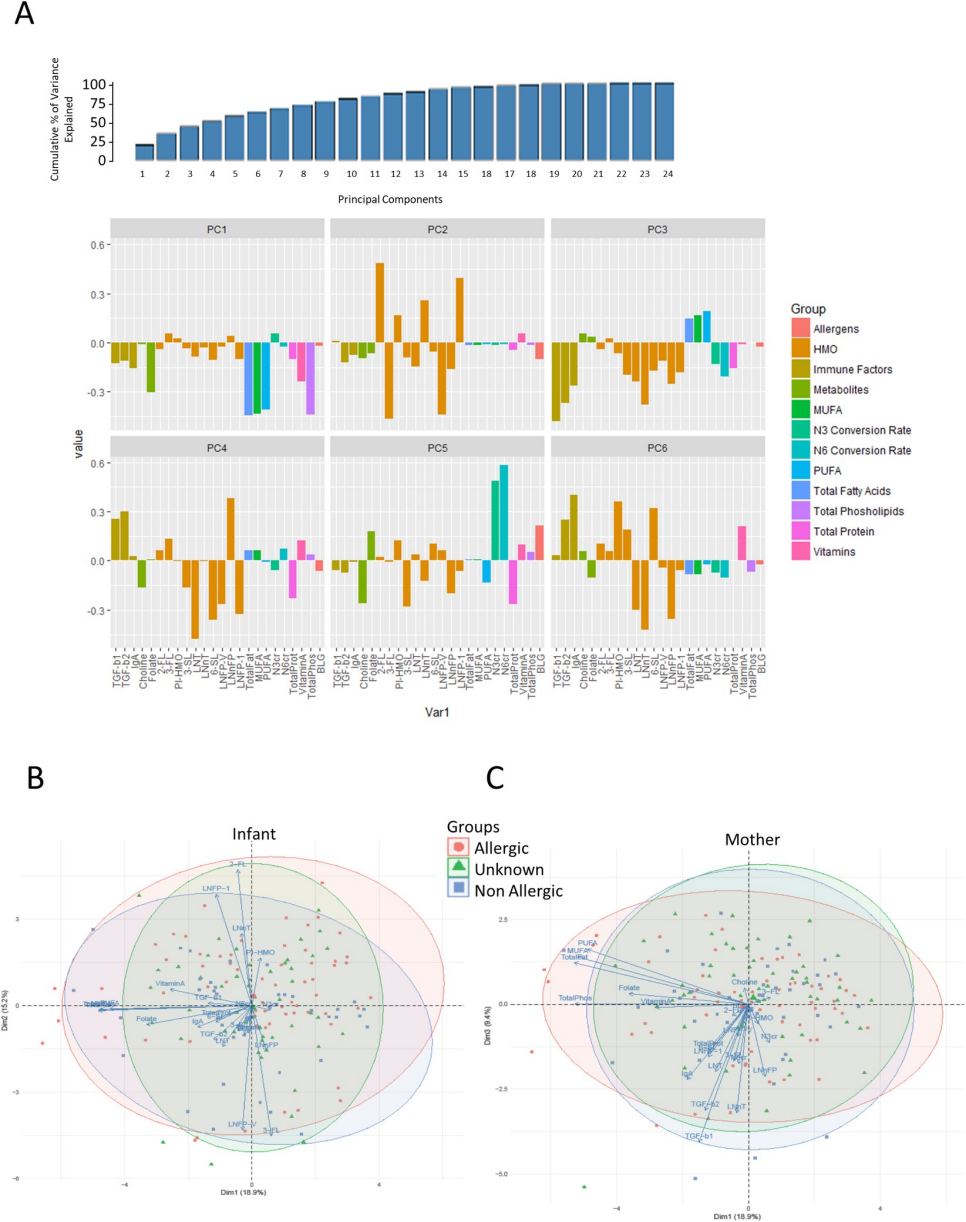
Principal components analysis (PCA). Amount and cumulative percentage of variance in each component in the PCA (A). PCA analysis with the allergic status in the infant (B) and the mother (C) is shown. The length of the arrow corresponds to the strength of the correlation between a variable and the PC axes. The direction of the arrow shows which direction and with which axis the variable (breast milk component) is correlated.

Principal component analysis (PCA) was then performed to obtain a lower-dimensional representation of the data, and to investigate the composition in the context of allergic diseases and maternal or child sensitization. The cumulative percentage of variance captured in the first five principal components was <60%, highlighting the complexity of the breast milk composition (Fig 2A). Lipid and protein variance were captured in the first principal component in the same direction on the y-axis. The HMO variance was captured on the second and third components. The HMO and immune factor variances were projected in the same direction on the third component axis. IgA was only well represented in the fifth component (Fig 2A). When observations were identified by maternal or child allergy status, no clear data separations of the groups were observed (Fig 2B and 2C).

### Visualization of the inter-dependencies between milk components

**Fig 3:**
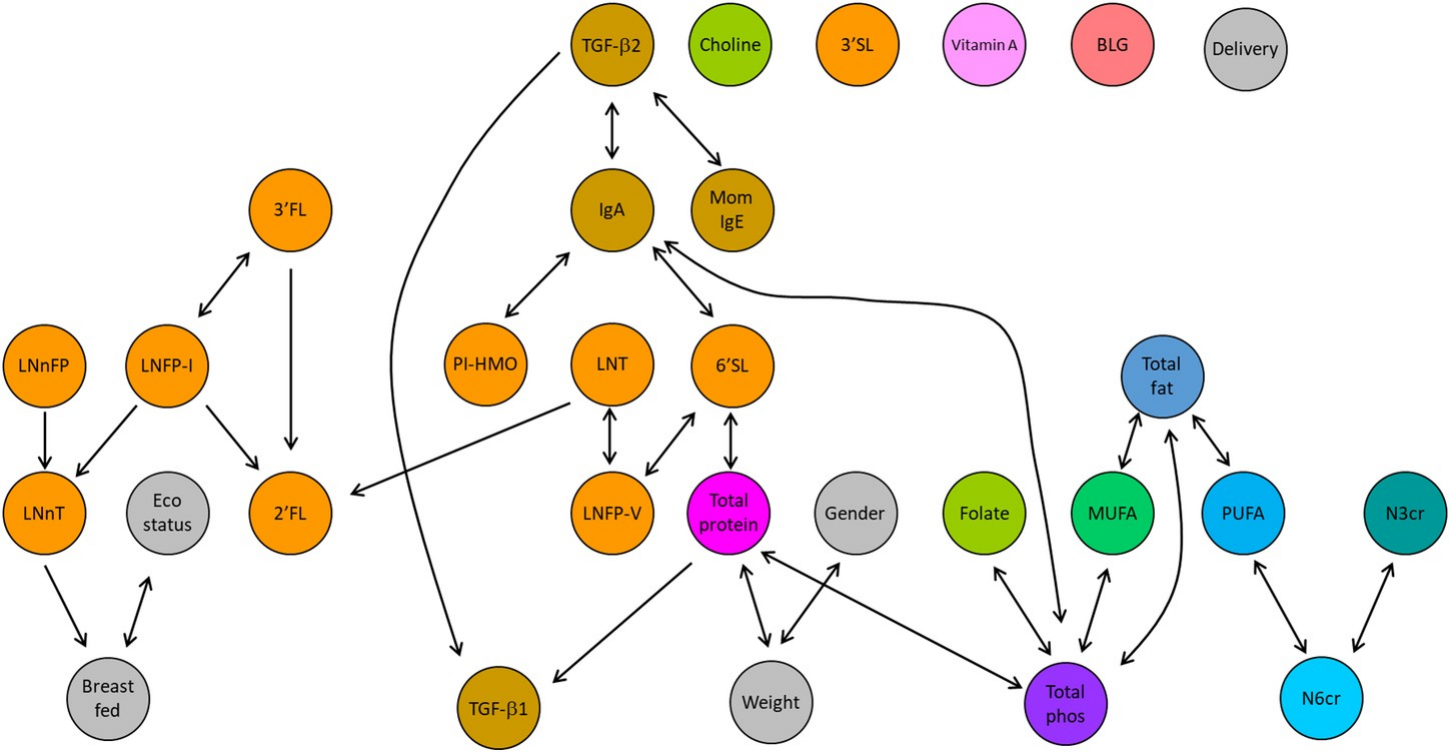
Graphical discovery analysis. An arrow pointing from a variable x to a variable y indicates the influence of the first variable on the second one. The length of the arrow and the position on the graph was generated automatically. The colouring of the components is similar to the one used in PCA. Possible confounders are in grey.

Finally, to visualize breast milk components and possible confounders inter-dependencies, Graphical Discovery Algorithm (GDA) based on causal discovery algorithm and graphical inference was used (Fig 3). The infant sensitization level was omitted as measured in less than 30% of the infant participants. This technique allowed the visualization of the inter-dependencies between component levels and the metadata that were not observed using the previous correlation matrix or PCA. Both presumed (based on prior literature) and novel interactions were identified with the GDA. Total phospholipids, IgA and total protein were the most connected components. Considering one arrow as one connection and a bidirectional arrow as 2 connections, total phospholipids, IgA and total protein displayed 10, 8 and 7 connections respectively. Interestingly, there was a unique dependency with maternal plasma IgE, connected directly with the immune markers TGF-ß2. The analysis also revealed a possible dependency between total protein and birth weight. It also draws attention to the apparent competition between the different fucosyltransferases for the fucosyl group to form 2’FL, 3’FL and LNFP-I. In contrast, 3’SL was not connected with any other HMO, and A-tetrasaccharide (PI-HMO, a blood group A marker) was only connected with IgA. Novel dependencies between IgA levels and 6’sialyllactose (6’SL) as well between 6’SL and total protein were identified. Other unexpected findings include the dependency between LNnT levels and exclusive breastfeeding duration (breastfed). Altogether, these data suggest inter-dependencies between maternal sensitization and the breast milk immune component TGF-ß2; TGF-ß2 with IgA and IgA with specific human milk oligosaccharides more specifically 6’SL, which itself is linked with total protein, and then indirectly to the other HMO levels measured in the LIFE Child cohort.

## Discussion

Overall, our study represents an original first attempt to visualize the complexity and inter-dependency of breast milk components. Our results illustrate the complexity of the breastmilk composition at 3 months postpartum in the LIFE child cohort. This is exemplified by the limited percentage of the variance that could be captured in the first 3 components of the PCA (less than 60% was captured within the 5 first components) and the numerous inter-dependencies identified in the graphical discovery analysis. Yet, the correlation matrix and more importantly the graphical discovery analysis isolate the possible inter-dependencies ruling the breast milk component expression in this cohort.

Numerous immunological components in breast milk have been associated with allergy risk in the infant [27]. Our data draw the attention on the inter-dependency between IgE in the mother plasma and the immune marker TGF-β2 and indirectly with IgA and TGF-ß1 in the breast milk. This finding partially validates our model as it identified a cluster of interactions (IgA, TGF-ß1, and TFG-ß2) that was already suggested in other studies [9–11]. Yet most studies, have looked at the influence of breast milk on the offspring allergy rather than the influence maternal sensitization could have on breast milk composition. Maternal allergy is one of the main risk factors of allergy risk in the infant. The possibility that maternal sensitization influence breast milk components directly or indirectly add a new perspective on the understanding of possible hidden interactions.

It remains unclear in the scientific literature whether specific HMO are more protective against allergy than other components. A recent paper by Miliku *et al*. investigated the relationship between 19 HMO and the development of food sensitization in infants [16]. Using multivariate analysis, they reported no positive or negative association between sensitization and individual or total HMO but that overall HMO profiles differed significantly between sensitized vs. non-sensitized infants [16]. Another study by Seppo *et*. *al* also reported that breastfed infants consuming breast milk with low concentrations of 6’SL, LNFP-III and LNFP-III were more likely to develop cow’s milk allergy but were not necessary for protection and concluded that other substances or mechanisms must be in play [14]. In the present study, the data are suggesting possible indirect influences of allergic sensitization in the mother (based on IgE level) on HMO in breast milk (indirectly via TGF beta2 and IgA). Some key confounders to several HMO levels have been identified and include seasonal and geographical variations along with other possible associated factors such as parity, ethnicity and breastfeeding exclusivity [28]. This last finding is quite interesting since our data also suggest a link between exclusive breastfeeding duration and LNnt levels whether this dependency or others identified here may be representing new physiological processes will need to be further tested.

To our knowledge, our data represent the first attempt to characterise the complexity of breastmilk using visualization of the multivariate dependencies between some breast milk components and their confounders. No associations with allergy in the infant could be found. Yet, our findings shed light on the complex inter-relationships between breast milk components. We also identified a dependency between breast milk TGF-β2 and maternal sensitization based on IgE level in mother blood as well as between breast milk IgA and PI-HMO and 6’SL. Some limitations are inherent to the design and the technique used. To consider only bivariate (and not partial) correlations in such settings may lead to spurious findings at the level of the p-values since the overall data structure is not considered. This is the reason why both r and p-values are considered rather than p-value only. The purpose of the multivariate analysis (the PCA and the GDA) was exactly to overcome this situation of false-negative p-value in correlation matrix, by exploring the dependencies between variables. Indeed, since PCA and GDA rely on the Pearson correlation matrix between variables; we only presented the Pearson correlations in order to be coherent with multivariate methods because considering the ranking of the data rather than the levels is less relevant in PCA or inference analysis as the dynamic of the data distribution is lost. Here we were not able to identify a separation of the data with the allergic status in the mother or the mom based on PCA. It is important to keep in mind that separation of data using PCA need to be interpreted with caution as PCA is intended to provide a lower-dimensional representation of the data by capturing the maximal variance of the data into each component and are not intended to identify significant differences between groups [29]. One limitation here is that PCA relies on some kind of linear assumptions and that any non-trivial association with the response would not be spotted with PCA alone. Elastic net and other methods of variable selection [30] are very powerful techniques for selecting factors associated with the response, however, the goal here was to explore different ways of visualization of complex relationships and not modelling them directly reason why emphasis on GDA was made. It must be noted that employing a multivariate approach is not without limitations such as false-positive discovery. In addition, breast milk is a dynamic fluid, and the small sample size could have influenced these data. Further research at different time points, in larger well-controlled cohorts, and standardized sample collection are required to confirm the validity and relevance of this new approach. Our sample size may in part be an explanation behind the absence of separation between the groups.

In conclusion, this study highlights how an original multivariate approach can be used to unravel the complexity of breast milk composition. It also suggests that conducting univariate and multivariate analyses in parallel may address some of the literature inconsistencies from studies that have examined breast milk components and their positive or negative associations with health outcomes such as allergy.

## Supporting information

S1 Fig Flow chart and characterization of the patients. (TIF)

S2 Fig Pearson correlations. Pearson correlation represented by the color gradient based on the r coefficient values for the 51 components and 2 composite ratios (A and C). Pearson correlation represented by the color gradient based on the r coefficient values with p-values based on Pearson correlation test (text values), p-values are rounded to two digits, meaning that a 0 value represents a p-value less than 0.01. P-values are shown for the 24 selected breast milk components (B).
(PDF)
