# Supplementary figures and images for: Novel approach to visualize the inter-dependencies between maternal sensitization, breast milk immune components and human milk oligosaccharides in the LIFE Child cohort
Source: PLoS One. 2020 Apr 21;15(4):e0230472. doi: 10.1371/journal.pone.0230472 (PMC7173766; doi:10.1371/journal.pone.0230472)

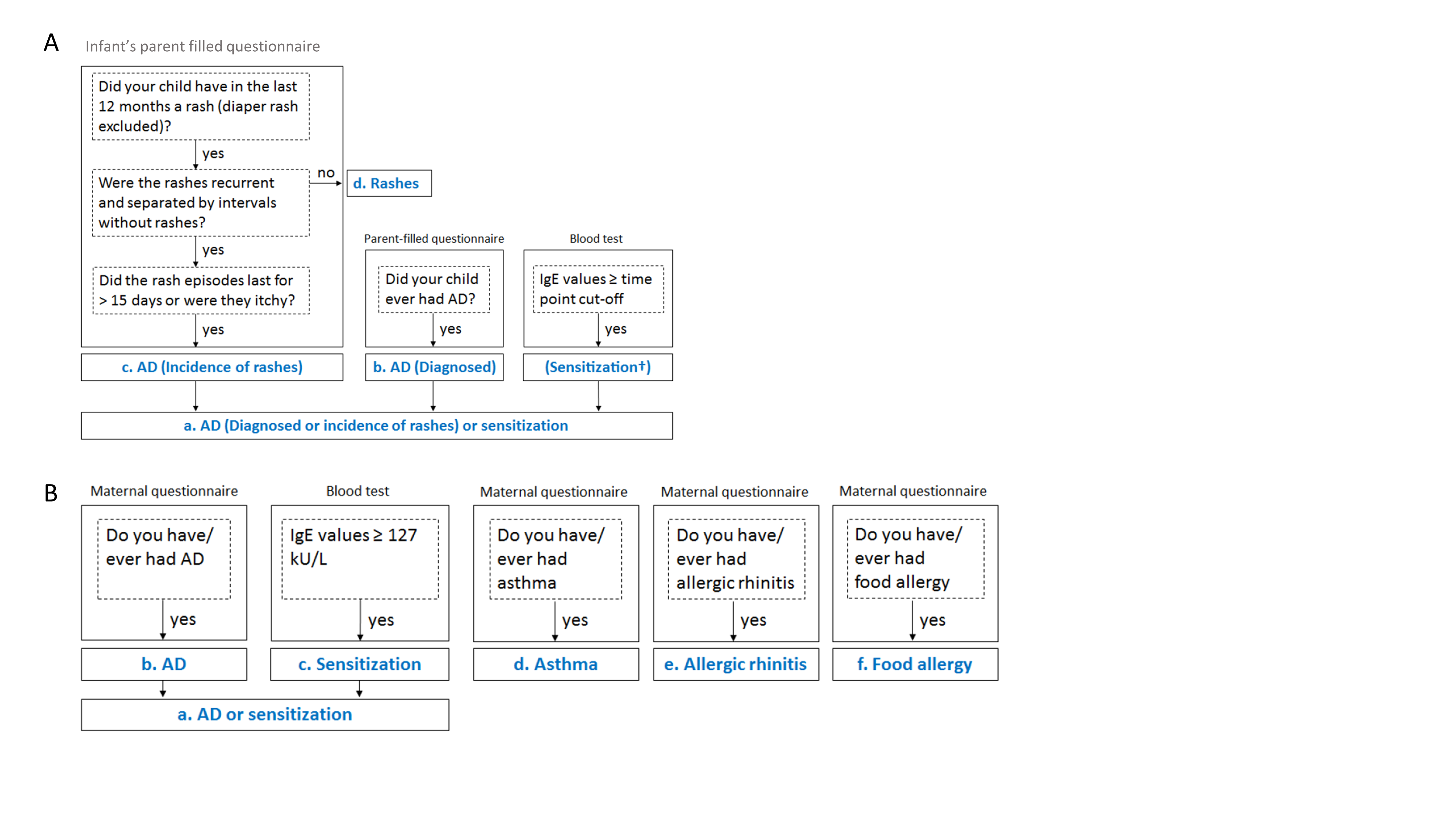

Supplement: S1 Fig — (TIF) [file pone.0230472.s001.tif]

A

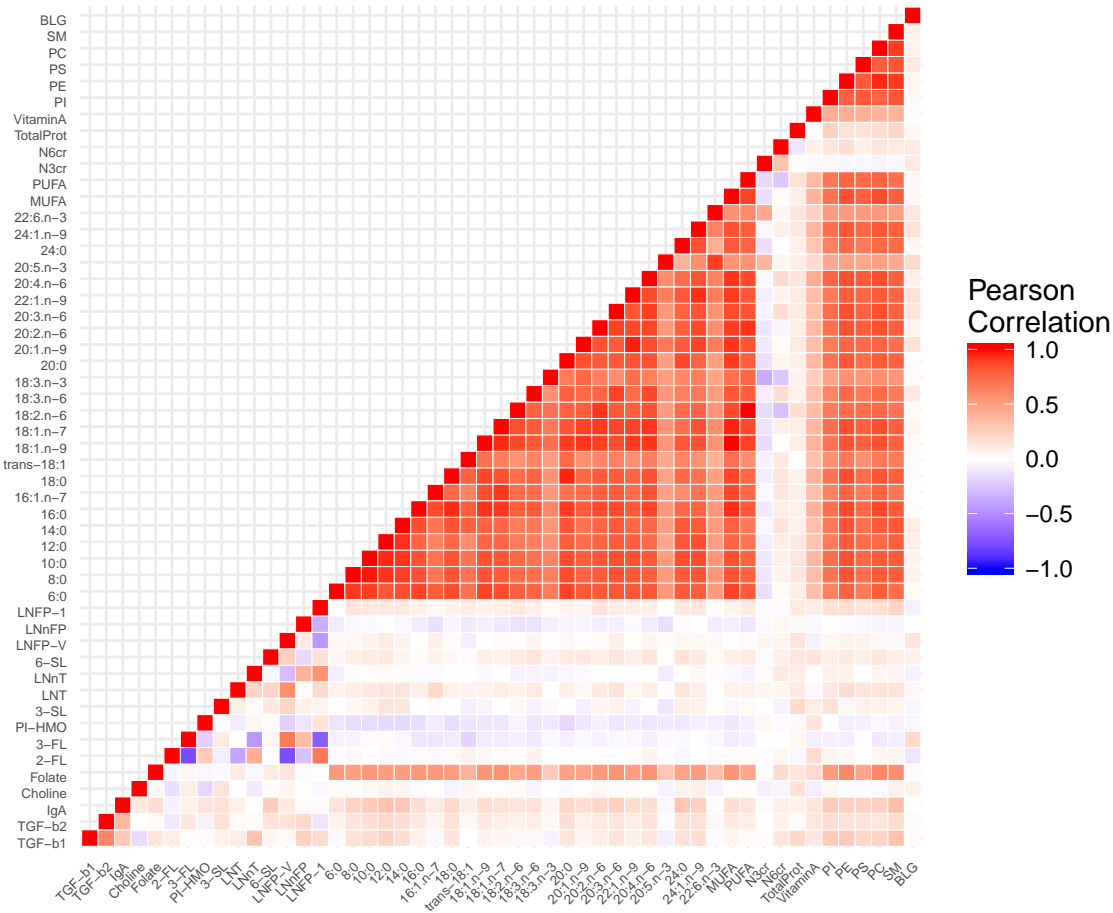

B

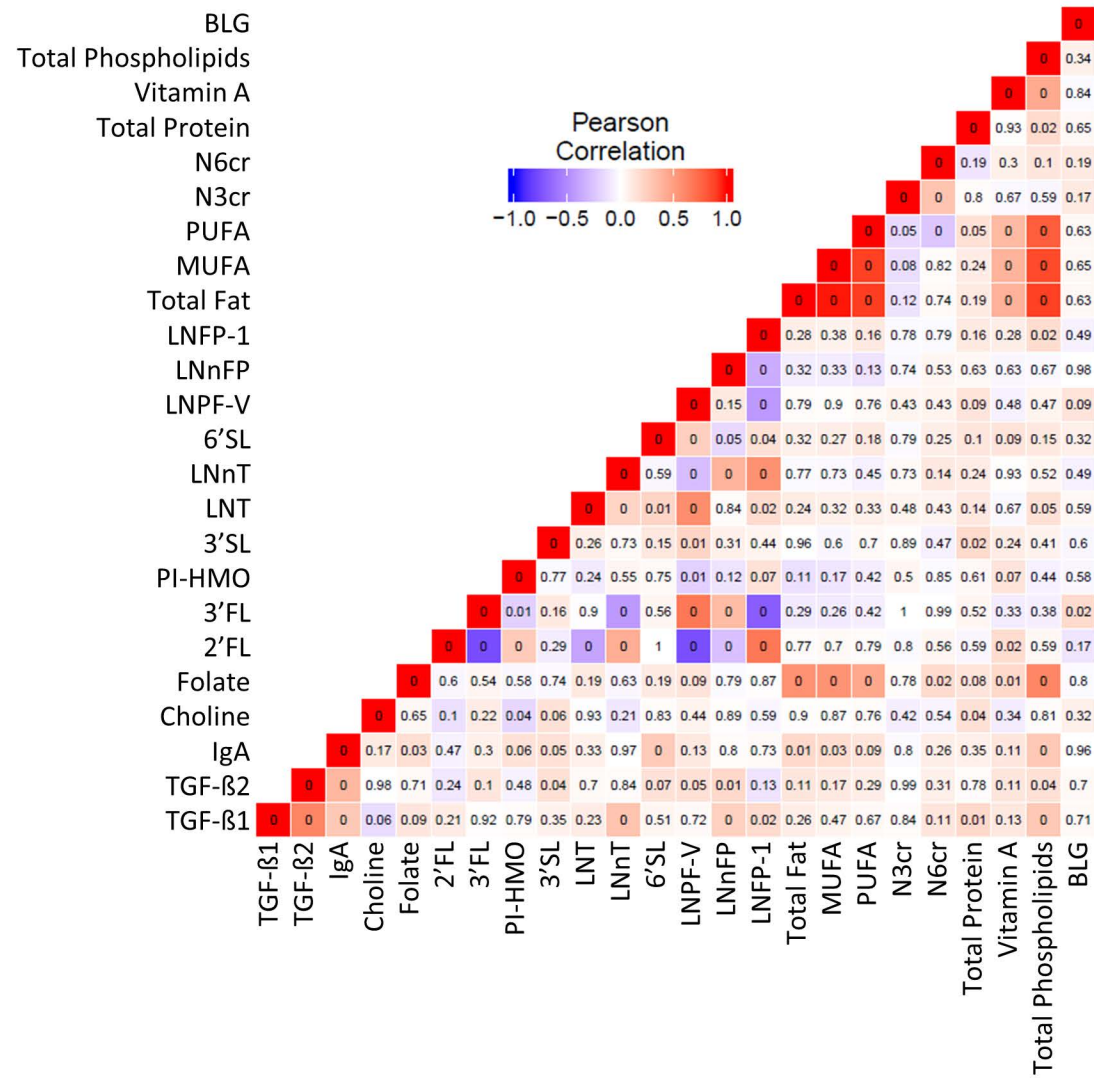

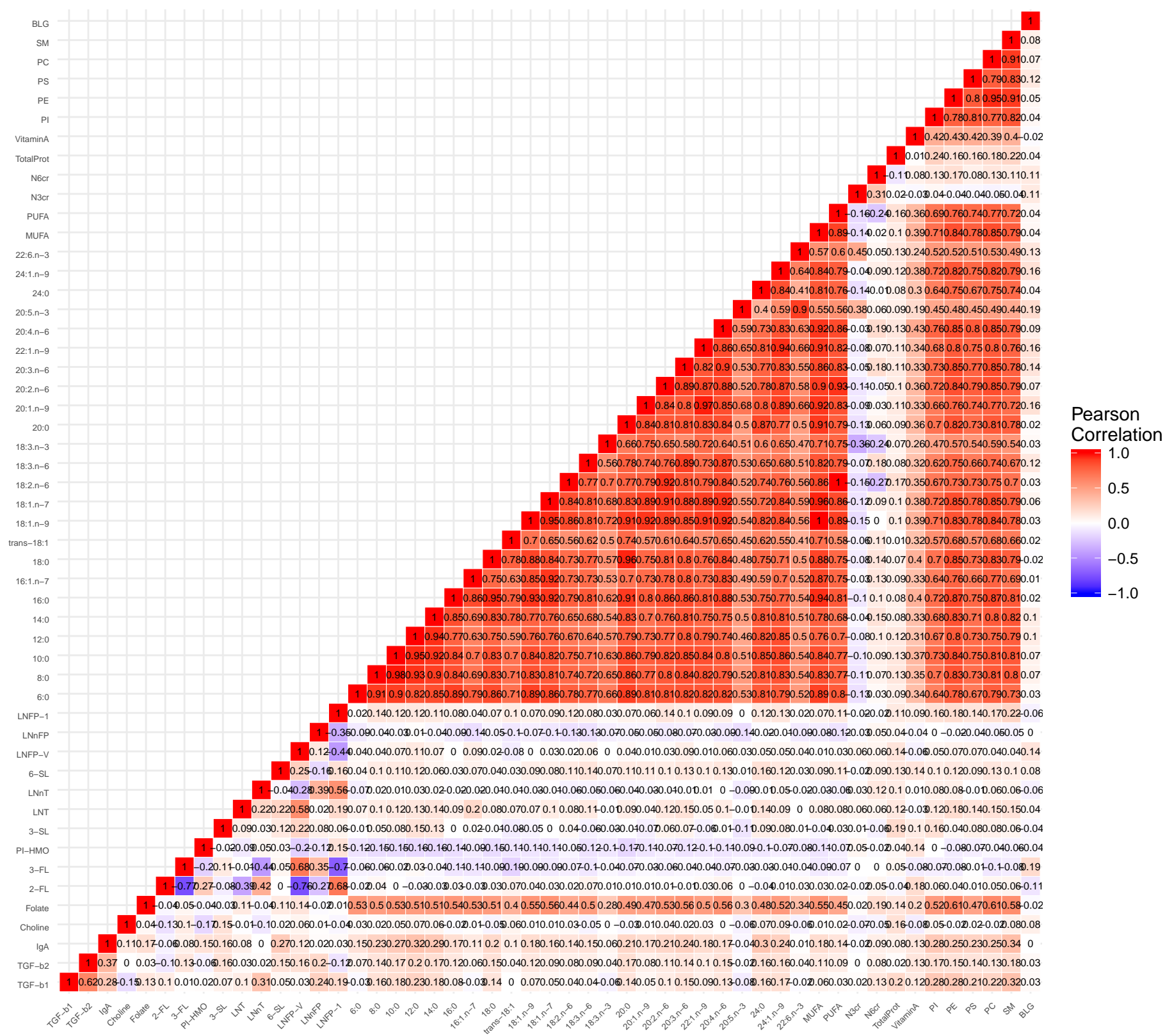

Supplement: S2 Fig — Pearson correlation represented by the color gradient based on the r coefficient values for the 51 components and 2 composite ratios (A and C). Pearson correlation represented by the color gradient based on the r coefficient values with p-values based on Pearson correlation test (text values), p-values are rounded to two digits, meaning that a 0 value represents a p-value less than 0.01. P-values are shown for the 24 selected breast milk components (B). (PDF) [file pone.0230472.s002.pdf]
